# Beyond mapping: a case for geospatial analytics in humanitarian health

**DOI:** 10.1186/s13031-019-0234-9

**Published:** 2019-11-08

**Authors:** P. Gregg Greenough, Erica L. Nelson

**Affiliations:** 1 Department of Global Health and Population, Harvard T.H. Chan School of Public Health, 677 Huntington Avenue, Boston, MA 02115 USA; 2 Harvard Humanitarian Initiative, 14 Story Street, Cambridge, MA 02138 USA; 3 Department of Emergency Medicine, Harvard Medical School, Boston, MA 02115 USA

**Keywords:** Humanitarian health, Geospatial analysis, Spatial analysis, Geographic information systems, GIS

## Abstract

The humanitarian sector is increasingly adopting geospatial data to support operations. However, the utilization of these data in the humanitarian health arena is predominantly in thematic map format, thereby limiting the full insight and utility of geospatial information.

Geospatial analytics, in contrast, including pattern analysis, interpolation, and predictive modeling, have tremendous potential within the field of humanitarian health. This paper explores a variety of historical and contemporary geospatial applications in the public health and humanitarian fields and argues for greater integration of geospatial analysis into humanitarian health research and programming. From remote sensing to create sampling frames, to spatial interpolation for environmental exposure analysis, and multi-objective optimization algorithms for humanitarian logistics, spatial analysis has transformed epistemological paradigms, research methods and programming landscapes across diverse disciplines.

The field of humanitarian health, which is inextricably bounded by geography and resource limitations, should leverage the unique capacities of spatial methods and strategically integrate geospatial analytics into research and programming not only to fortify the academic legitimacy and professionalization of the field but also to improve operational efficiency and mitigation strategies.

## Background: geospatial tools in the humanitarian space

We all live in a world of spatial data. Humanitarian actors in particular work in a space where population migration and dynamic political boundaries are the norm. One would argue they are awash in information that defines and elaborates the space of a crisis. Logisticians characterize areas most affected or insecure and optimize supply lines. Water and sanitation engineers delineate coordinates of water tables, envision distribution lines and plot water delivery points. Food security agencies develop models to streamline the route between food sources and affected-populations. Shelter managers digitize the ebb and flow of displaced populations, and health providers map facilities, disease incidence and care delivery capacities. These time-space factors are increasingly emphasized through geospatial frameworks to advance operations. However, the predominant use of spatial data in humanitarian health is for thematic mapping, which limits the potential insights and full utility of geospatial information. The intentional, systematic incorporation of geospatial analysis with its advanced forms of cluster analysis and predictive modeling is critical for transforming the humanitarian response field, academically and programmatically.

Thematic maps have long been used to inform humanitarian actors regarding the location of affected populations, access routes and contextual topography. The visual representation of data transcends linguistics and creates an efficient and effective mechanism supporting preparedness, response and coordination. But geospatial sciences have evolved from basic cartography into a field of geo-statistics, modeling and interpolation that takes points, lines, and polygon areas with attributes in space, and revamps them into powerful models with predictive, hypothesis-testing and inferential capacities. And while operational mapping has day-to-day utility for humanitarians, the analytic power of geospatial information can be transformative for a wide range of disciplines.

**Fig. 1:**
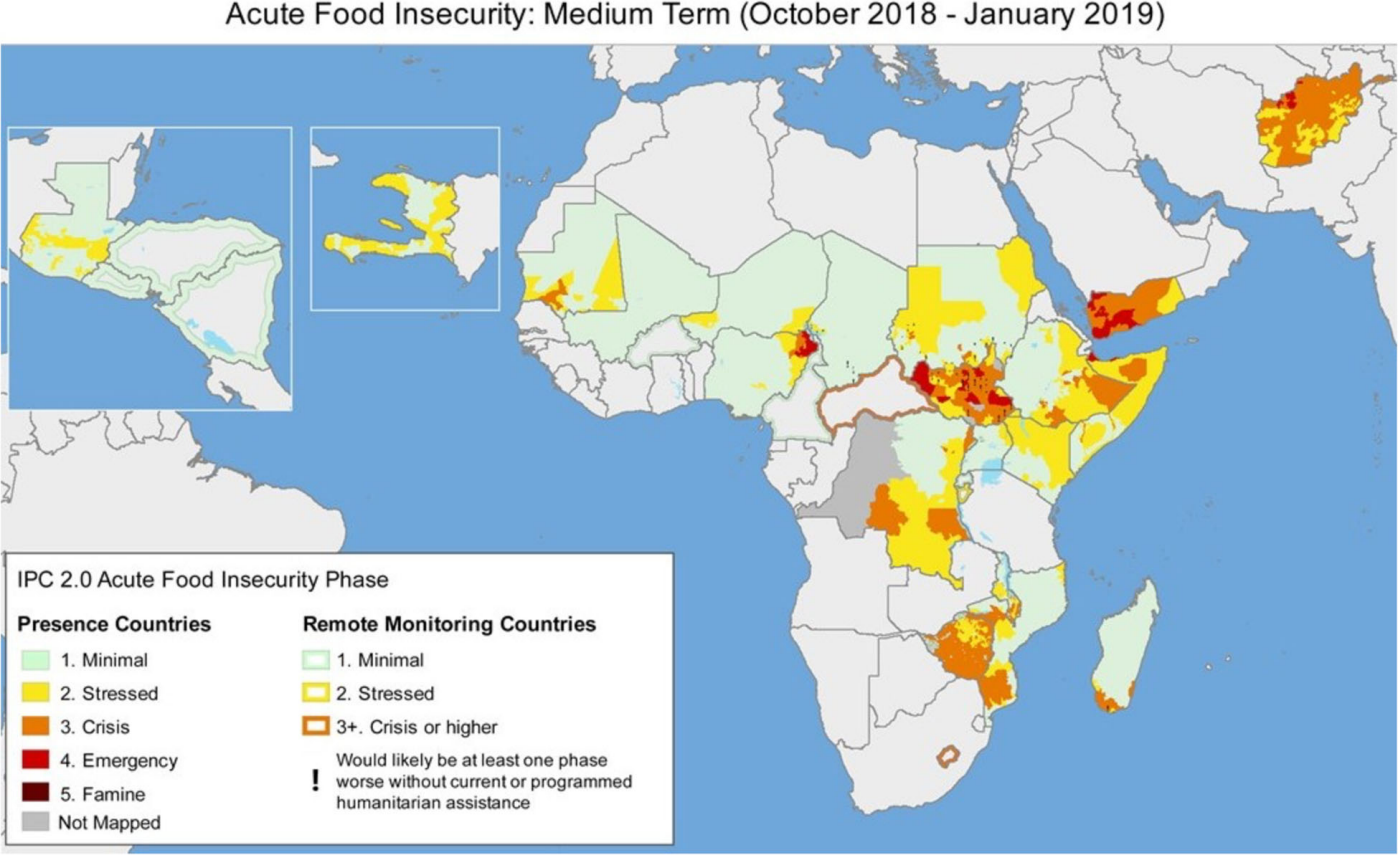
Contemporaneous thematic map of food security, FEWS-Net. The Famine Early Warning Systems Network coalesces prospective food security data in a mapped format to indicate geographic areas of acute food insecurity by degree of severity. Used unedited with permission from the Famine Early Warning Network

Geographic Information Systems (GIS) are the tools designed to capture, store, manipulate, manage and visualize spatial or spatiotemporal data. GIS as a fundamental technology in all types of humanitarian crises, is largely accepted and integrated into response operations. Thematic maps, with relevant attributes layered onto topology, continue to be useful in all of the aforementioned programmatic sectors to identify and target interventions. Initiated in 1985, one of the earliest and now familiar mapping products that humanitarians have relied upon is the Famine Early Warning System Network (FEWS-Net), a venerable stream of mapped food security indicators, gathered and synthesized across global agencies [1]. While thematic, these maps provide predictive data and inform not only the food security sector, but also other sectors impacted by food, namely health and nutrition (Fig. 1). Others use spatiotemporal diagrams to document and visualize gross human rights violations during conflict, advocating without statistical inference for the protection of civilians and humanitarian space [2]. Agencies tasked with civilian protection employ spatial modeling of satellite data to analyze a range of population dynamics from settlement activities to behavior patterns of militant groups [3].

Experts in the field of humanitarian forensics are also thinking spatially, applying geo-statistical models for grave site locations and missing persons searches [4]. Regression techniques that utilize location as a critical variable are being applied to predictive landmine risk mapping and provide validated locations to prioritize demining operations [5]. All of these illustrate geospatial applications to highlight populations at risk and to trigger protective policies.

**Fig. 2:**
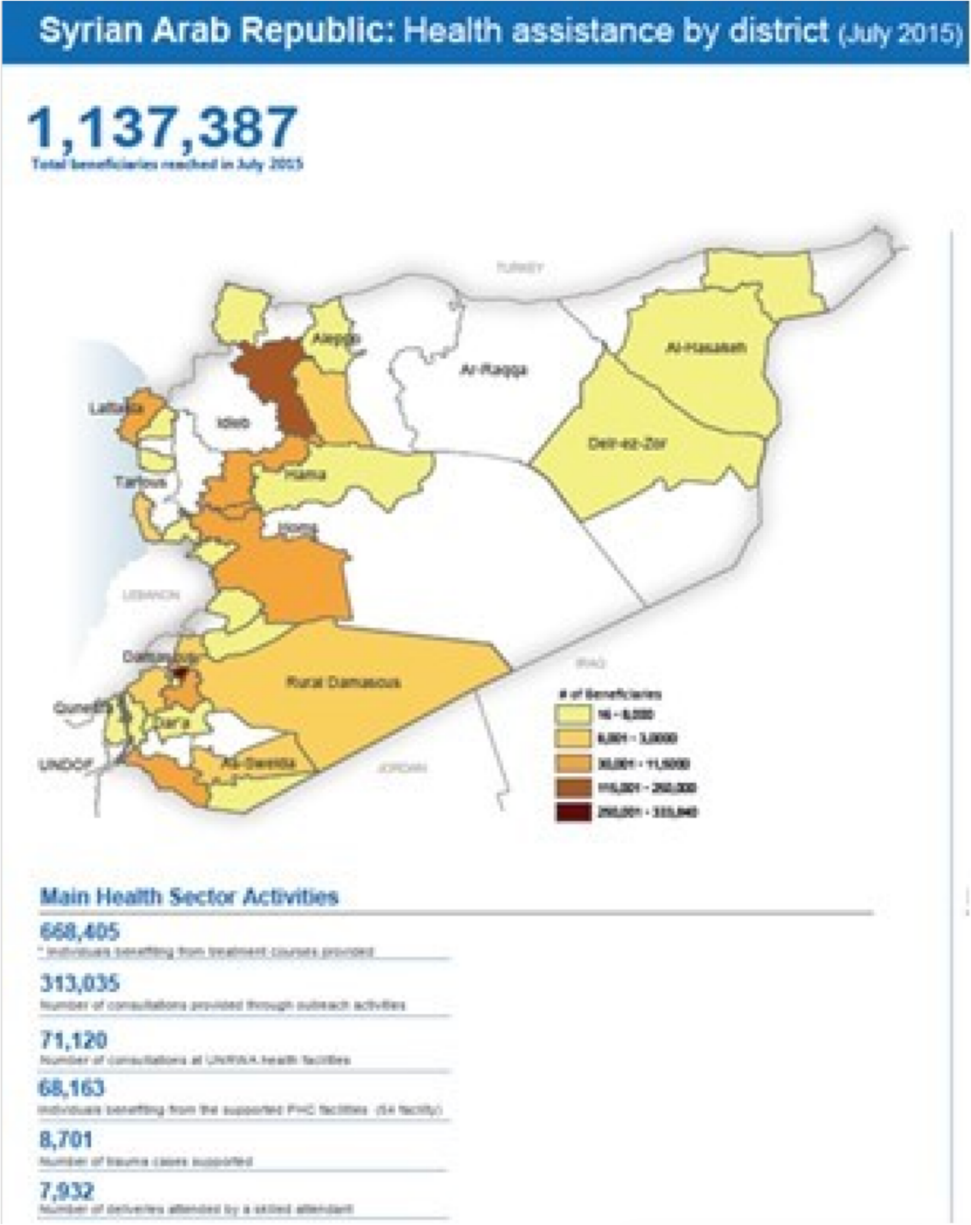
OCHA-generated health sector operations map within Syria. The choropleth map shows the density of beneficiaries in relationship to health sector activities. Used unedited with permission of the UN Office for the Coordination of Humanitarian Affairs

**Fig. 3:**
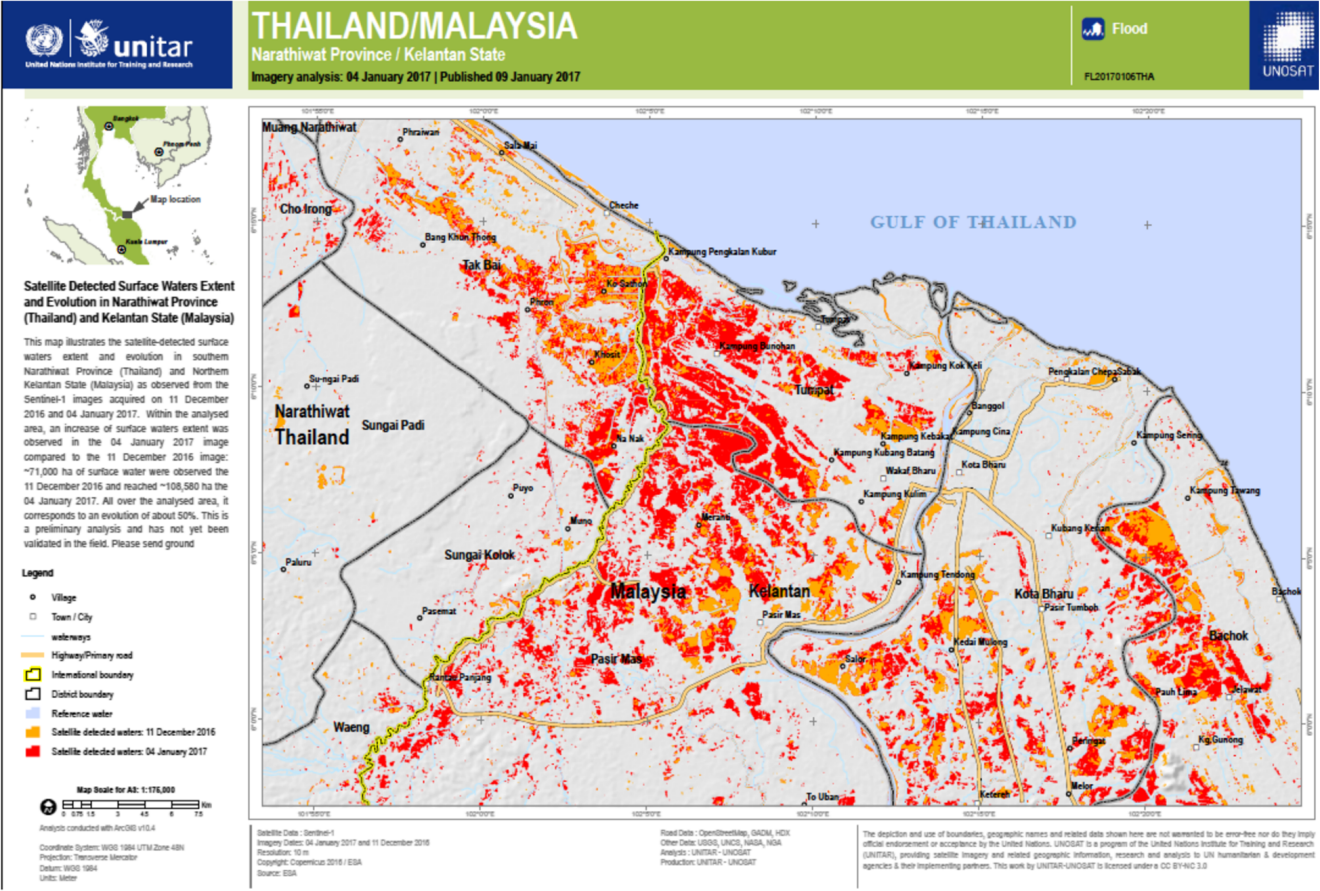
UNOSAT-generated Thailand-Malaysia border flood situational report for OCHA’s humanitarian information unit. This remotely sensed image analysis map product from two satellite images taken 11 December 2016 and 4 January 2017 shows color-enhanced areas of increasing flood zones in relation to human settlement areas. Used unedited with permission of the UN Office for the Coordination of Humanitarian Affairs

The fundamental ‘everything happens in time and space’ principle is informing the work of the UN Office for the Coordination of Humanitarian Affairs (OCHA). Tasked with coordinating a vast number of humanitarian stakeholders and managing exorbitant amounts of information from unilateral, multilateral, local, and multi-sectoral stakeholders, OCHA now routinely produces thematic maps and infographics to support coordinated decision-making. This seasoned operational agency serves as an open repository of graphic outputs, interactive tools, common operational (including geocoded) datasets across all sectors, and shared maps and rapid assessment data that describe ‘who is doing what and where’ [6]. Figure 2 is an example of operational data for the health sector within Syria. UNOSAT, the Operational Applications Satellite unit within UNITAR, the UN Institute for Training and Research, provides satellite image analysis and map products for humanitarian operations such as OCHA’s situational reports (Fig. 3).

The result is enhanced coordination. While maps have facilitated much of humanitarian history, the geospatial renaissance in which shared spatial data is the fulcrum for enhanced coordination was largely driven by voluntary organizations with GIS skills after the 2010 Haiti earthquake. Non-governmental information and communication-focused groups, despite being new to the world of humanitarian operations and coordination, coalesced around harnessing crowd-sourced geospatial data in support of targeted relief [7]. These crisis mappers advanced open platforms and shared data across sectors and stakeholders, developed application programming interfaces (APIs) for new mapping and analytic programs, and evidenced the potential of operational cartography and geospatial analytics drawn from sources at all levels, disciplines and experience.

Humanitarian logistics –whether focused on supply chains, resourcing or distribution, leads the incorporation of geoanalytics into decision-support tools for humanitarian operations [8]. Multistep models, informed by operations research methodology, integrate remotely-sensed (either aerial or satellite) imagery, land use, road maps, land elevations and pre-existing infrastructure to determine cost surfaces and risk profiles. Multi-objective optimization models seeking to minimize travel distance, risk along evacuation paths, time in transit, and resource allocation utilize GIS to discriminate emergency facilities, shelter placement and evacuation routing in flood preparedness [9, 10]. The scope of geospatial data utilization within humanitarian logistics continues to expand from visualization and spatio-intuitive pattern recognition to situational and programmatic optimization, reducing risk for the disaster-affected and improving cost-effectiveness through empirical methodologies.

While other humanitarian stakeholders are making substantive use of geospatial data, the humanitarian health sector is lagging behind. The World Health Organization houses a map gallery within its Global Health Observatory but hosts no exclusive geospatial data on health in crises environments. Its collaborating center for geospatial analytics at the University of Oxford has a disease-specific focus (malaria), and although relevant to crisis-affected populations, the spatial analysis of this disease is contextualized predominantly in non-crisis affected populations. Forays into spatial models for humanitarian health exist, but generally, thematic maps continue to permeate its operational landscape, and a sophisticated integration of spatial analysis and GIS is lacking*.*

## Why geospatial analysis is good for humanitarian health

Disciplines such as urban planning, business and marketing, and a range of earth sciences have outpaced public and humanitarian health sciences in applying GIS. Two notable exceptions within public health—environmental health and infectious diseases—have embraced the power of geo-analytics to model health impacts for populations [11]. And disaster sciences, largely excluding disaster medicine, are beginning to incorporate remote sensing technologies for situational awareness and population enumeration. Mining the use of geospatial methods in these fields illustrates their significant potential to produce alternate ways of thinking that protect populations, build academic rigor, and improve programmatic efficiency in the humanitarian health sector.

### Thinking geospatially

Thinking geospatially allows us to ask and answer novel questions that illuminate our universe in ways distinct from those derived from other cognitive forms. Spatial thinking, as defined by the National Research Council, incorporates three elements into problem solving: concepts of space, tools of representation (e.g. the basis of coordinate systems and spatial projections), and processes of reasoning (e.g. definitions of proximity, extrapolation and interpolation, etc.) [12]. Repositioning data into a spatial frame changes the way we define questions, express and analyze relationships, transform and communicate information, and ultimately make decisions.

Space is thus a framework for understanding. Integral to human experience and evolutionary fitness, spatial thinking utilizes deeply ingrained, a posteriori cognitive models to explore data previously locked in static, analog mediums [13]. With the evolution of visual media, geo-tagged applications and virtual reality, people are accumulating even more intuitive spatial capacity. Once harnessed and honed, spatial thinking can function in a descriptive, analytic or inferential manner.

**Fig. 4:**
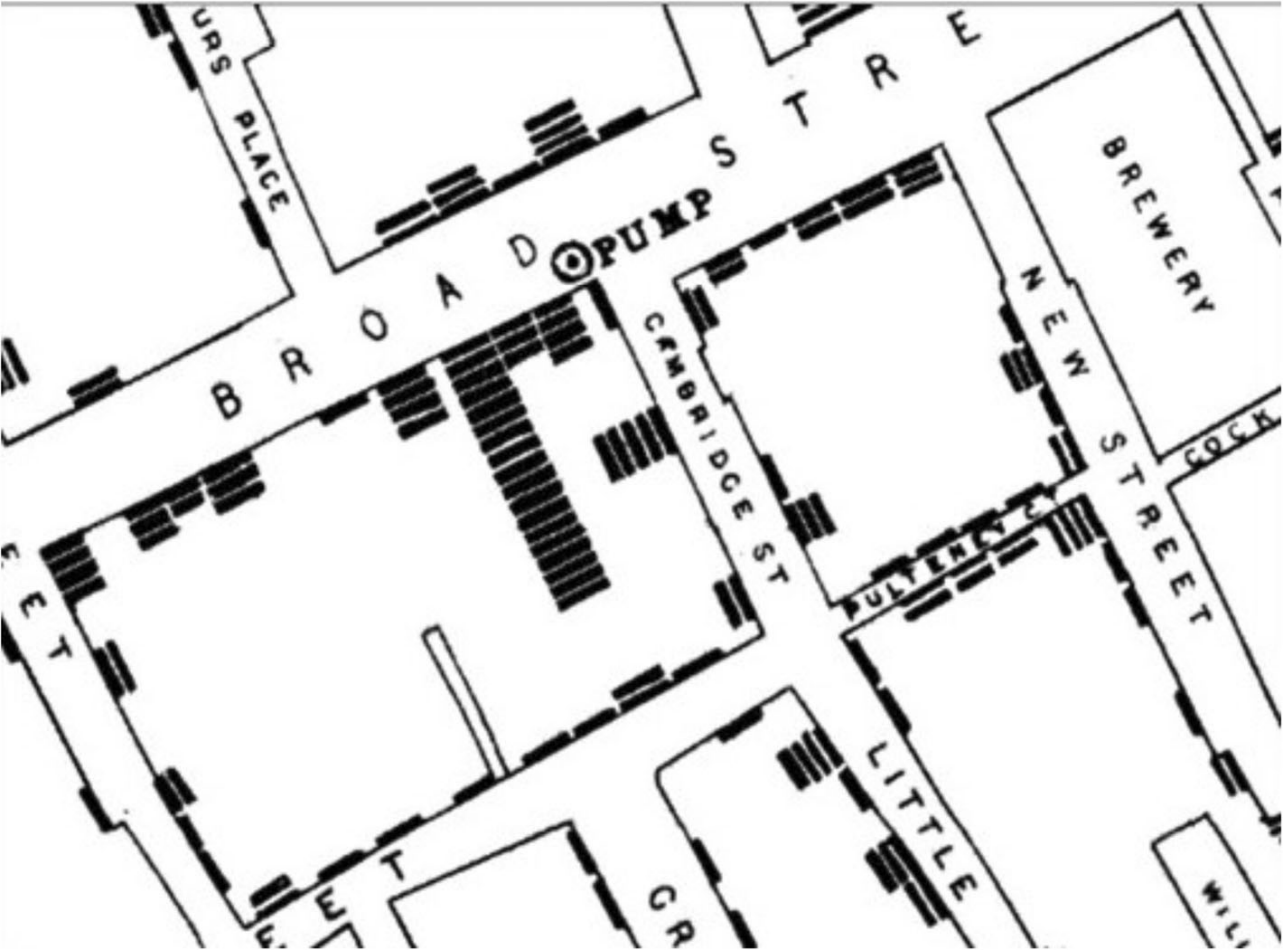
Map of the 1854 Cholera outbreak in relation to the water supply. John Snow’s mapping of cholera cases demonstrates the spatial approach to the science of public health, the dispersion and clustering of cases and their relationship to a causative agent. Terms of use: this work is licensed under a Creative Commons Attribution Generic License. It is attributed to John Snow and the original work can be found here, https://www.ncbi.nlm.nih.gov/pmc/articles/PMC2250686/pdf/brmedj06236-0004.pdf

One of the earliest and most notorious examples of spatial thinking in public health is Snow’s investigation of cholera cases in 1854 London. By adopting a spatial framework with which to evaluate the cholera outbreak, Snow dispelled the miasma theory of disease states and rightly identified Soho’s Broad Street pump as the prime culprit (Fig. 4) [14]. Spatially representing cases lead to a visual representation of distribution pattern, the identification of clusters, and potentiated new hypotheses regarding etiologies of the disease.

The evolution of spatial thinking within public health from fundamental spatial descriptions to its higher analytic functions is epitomized by the last two decades in the environmental health literature, as mentioned earlier [15]. Distance-decay models, wherein ‘exposure’ to a toxic site is assumed to be inversely proportional to one’s distance from a toxic facility, and interpolation algorithms that predict unknown values at a specific location from equations that rely on the principle of spatial dependence, are being used to identify not only the source of environmental contaminants but also the exposure patterns of victims within the populations exposed and unexposed [16]. Both hinge on the concept of spatial autocorrelation, in which objects closer in spatial proximity are more likely to share attributes. With this conceptual tool in hand, one can easily think about the implications of health for populations in crisis, displaced, or on the move. Does proximity to certain places show a statistical likelihood for certain health outcomes? Might certain places have a demographic profile that is exposed to more negative health indicators for morbidity or mortality? The geospatial hypotheses are endless. However, spatial thinking, its theoretical frameworks and unique statistical methodologies, are glaringly absent from many public health and the vast majority of humanitarian and disaster studies programs. Just as we emphasize the scientific method and inferential statistics as crucial learning within the empirical academy, so too should we incorporate geospatial statistics into curricula to grow a generation of humanitarian health professionals that can capitalize on these powerful tools.

### Advancing academic rigor

Adoption of geospatial science will improve the evidence base for humanitarian health operations. GIS, along with global positioning systems (GPS) and remote sensing instruments, can strengthen quantitative studies in public health and disaster science through population enumeration, the creation of sampling frames, and a foundation for validating analytical processes. The critical incorporation of these methods into humanitarian health research and subsequent interventions will create a wealth of empirical evidence and spur a field of inquiry that further legitimizes humanitarian medicine within academia.

### Estimating populations

Population enumeration and density mapping of affected populations is critical to all phases of humanitarian response. In particular, they are integral to needs assessments and resource allocation, and requisite for monitoring and evaluation efforts as the denominator for interventional effects. Developed nations have robust census infrastructure to provide geocoded demographic statistics. But in the context of humanitarian crises that frequently occur in underdeveloped nations or that overtly damage the vital registration collection infrastructure, baseline census data are usually incomplete, outdated, not disaggregated by age or gender, or are lacking sufficient spatial resolution. During disasters and conflicts, human populations are particularly dynamic, rendering whatever preexisting estimates available even more inaccurate. Ground-based census counting is time- and resource-intensive, contingent on security and access, and dependent on statistically inferior convenience sampling and not validated demographic methods used in stable static populations [17].

Remotely sensed data, derived from unmanned aerial vehicles (UAVs), manned aircraft, and satellites, offer access to large and inaccessible landscapes and are becoming increasingly available with better spatial resolution and lower operational costs. Very high resolution (VHR) and multi-spectral satellite imagery has been adopted over the last two decades by a broad range of actors. Population estimation is being done in non-emergency settings and for monitoring population variation in internally-displaced people camps from Sri Lanka to Ethiopia to Haiti with varying degrees of precision [18–23]. Structures, such as tents, are identified through object-based image analysis, counted and included in equations that extrapolate population numbers. But while ripe with the potential to provide accurate denominators for research, there continue to be challenges to the accuracy of satellite-derived remotely sensed data, as affected communities move from organized settlements into informal urban environments, living in multi-storied, adjoined buildings and mixing with host populations.

With significant penetration of cellular phone ownership throughout the globe, pattern analysis and geospatial predictive analysis provide compelling options for population enumeration and tracking. After the 2010 Haitian earthquake, Digicel, Haiti’s cellular carrier, provided de-identified cell phone data to researchers who applied behavioral algorithms to cell phone locations. With fair accuracy, they tracked population movements to and from Port-au-Prince over time [24]. Unfortunately, delays in data sharing agreements, computational infrastructure construction and analysis resulted in a four-month delay of usable data intended for real time programmatic intervention.

**Fig. 5:**
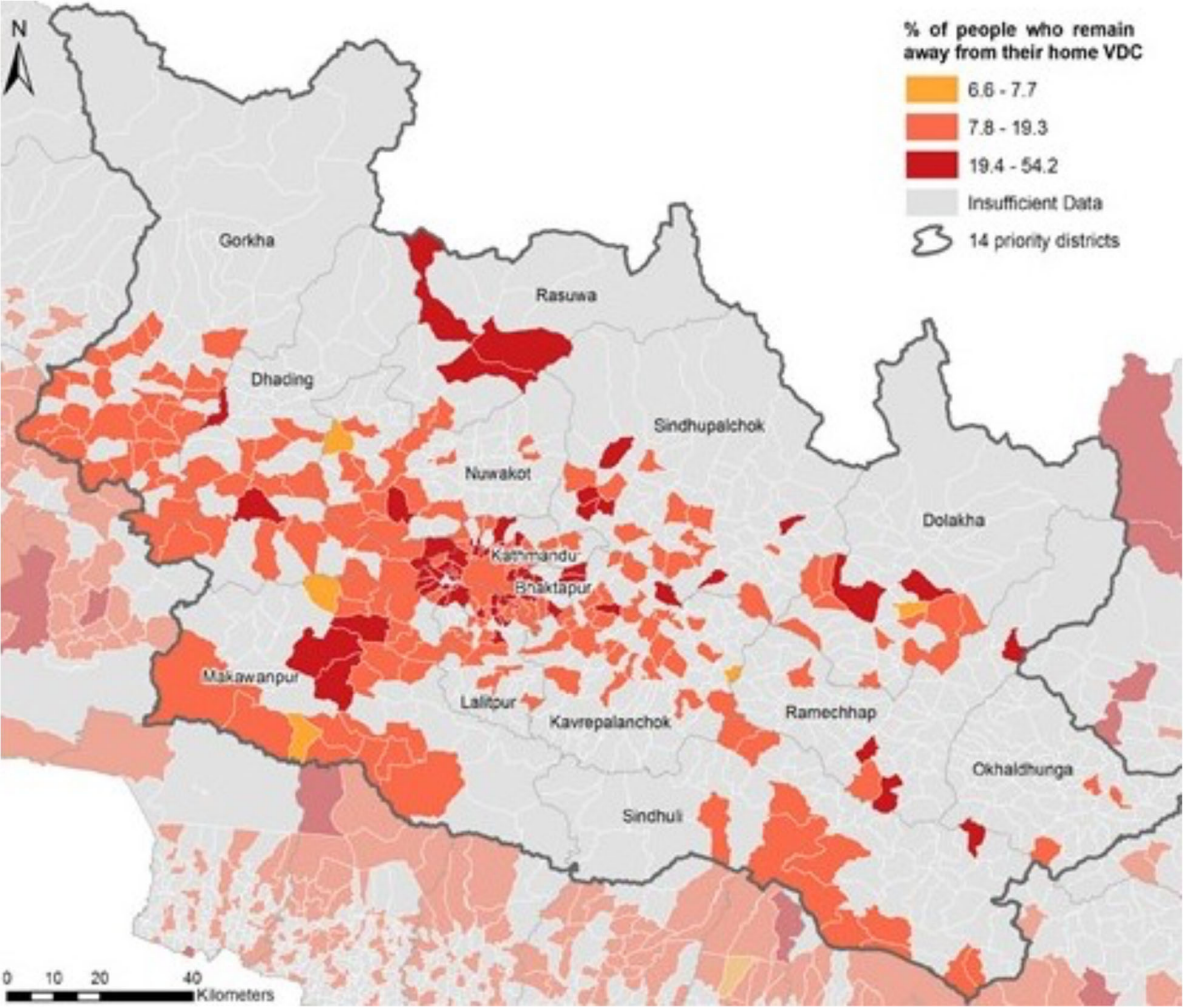
Spatial analysis of percentage of people who remained away from home, 4 months after the 2015 Nepalese earthquake. The map depicts call detail records at the sub-district or village development committee level (VDR) as a percentage of the population that remain displaced from their VDC of cell phone record 4 months after the earthquake. Terms of use: this work is licensed under Creative Commons Attribution License and used unedited. Attributed to Wilson, et al. PLoS Curr Dis, 2016; original version at: http://currents.plos.org/disasters/index.html%3Fp=27109.html

In contrast, in the immediate aftermath of the 2015 Nepalese earthquake, a fortuitous pre-arranged data sharing agreement with NCell and Telecom, the country’s two major cell phone carriers, allowed for a timely release of anonymized call detail records (CDR). Embedded within the CDRs was the point coordinates for the cell tower which were layered with gridded population data from WorldPop [25]. Incorporating temporal and spatial scales into a change matrix to delineate normal (pre-earthquake) population flows from post-earthquake flows, researchers could track population dynamics in real-time, 9 days after the event [26]. For the humanitarian health sector, an accurate understanding of population numbers and dynamics is critical not only for research but also for targeting resources (Fig. 5).

Now, efforts to use geo-tagged social media data to identify endogenous human behavior and track human movement are emerging, and analysts can differentiate the variance in human mobility at differing spatial scales [27]. There is even some evidence that spatial distribution data can be modeled using population-weighted models of opportunity to reflect city-scale human movement [28], a critical tool as humanitarian crises become increasingly urbanized. Thus, while there is tremendous potential for geospatial statistics to transform population enumeration and mobility tracking in complex environments, timely access to information through pre-identified data streams and pre-arranged data sharing agreements is crucial to the relevance of these modalities in the humanitarian space.

### Improving health survey sampling methods

Like many public health outcome measures, mortality and morbidity are critical indicators widely accepted by the humanitarian health community for understanding the health stressors on a population and tracking health impacts of operations. However, major challenges to implementing a randomized survey for health indicators exist. As discussed above, pre-existing population lists from which to generate a sampling frame and randomly select respondents suitable for statistical inference are often insufficient. Vital registration structures that record birth and death rates are destroyed or crippled in a crisis. Manually created clusters of geographic population units have been the historic default for researchers attempting randomized health outcome surveys. But this approach is time-intensive, requires significant ground-level mapping, risks selection bias, and introduces increased variance due to clustering effects depending on the health variable of interest.

**Fig. 6:**
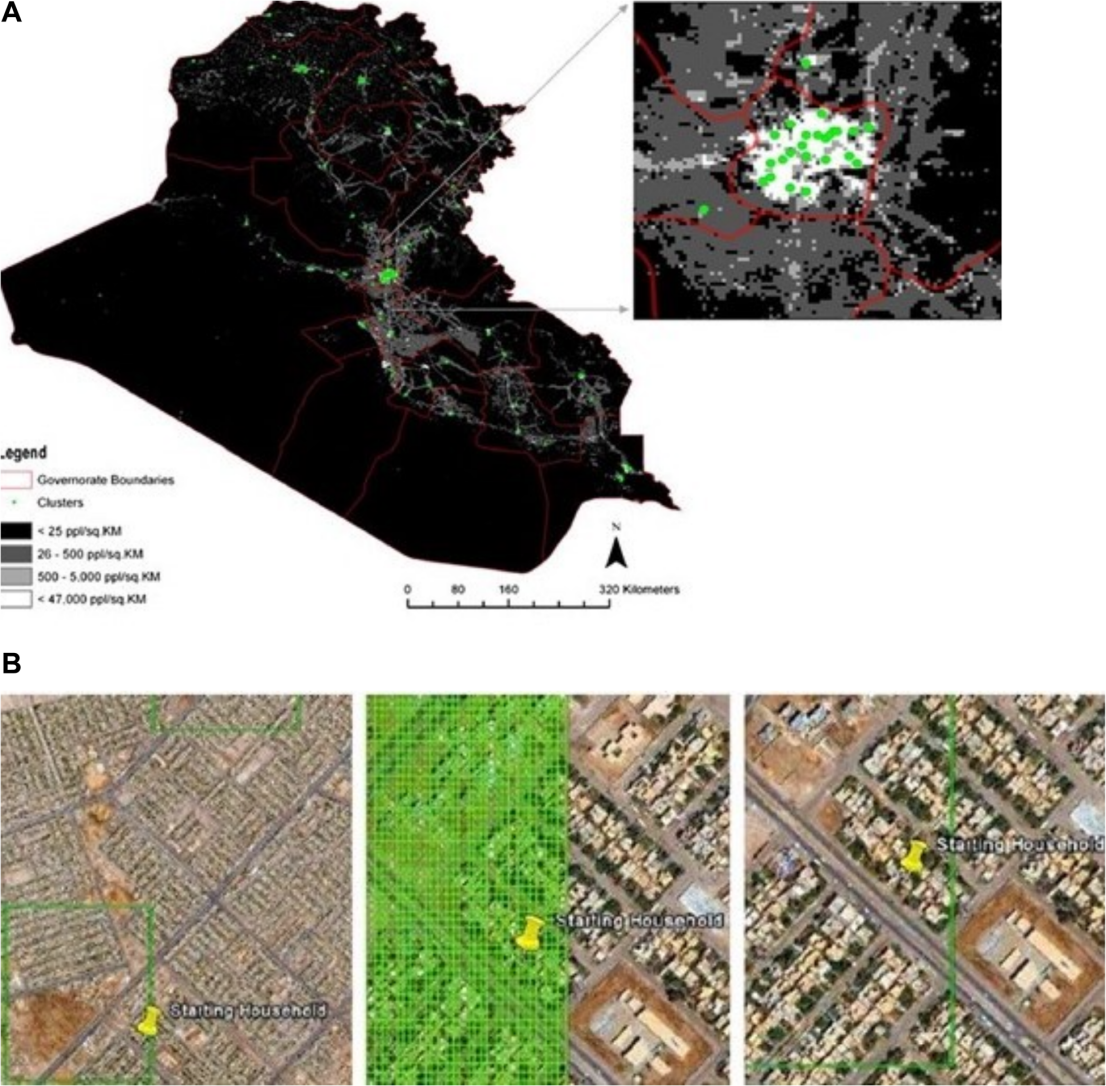
**a** (top). First stage of a population-based cluster sample using ArcGIS and LandScan, a gridded population dataset, *Galway,* et al. The main image frame (top) shows a LandScan (Oak Ridge National Laboratory) satellite image of Iraq with population density by square kilometer. The inset to the right amplifies these pixels of population density highlighting the more densely urbanized area of Baghdad. For sampling purposes, a population researcher can randomly select areas based on population dispersion. **b** (bottom). Second stage of sampling using ArcGIS grids on Google Earth kml files, *Galway,* et al. At the household sampling level, superimposed grids are randomly sampled using Google Earth imagery. Terms of use for both figures: this work is licensed under Creative Commons Attribution License 4.0 and used unedited. Attributed to Galway, et al., Intl J Health Geogr, 2012; original version at: https://ijhealthgeographics.biomedcentral.com/articles/10.1186/1476-072X-11-12

Remote sensing imagery, gridded population datasets, and spatial sampling algorithms are making this exercise far more efficient, and in the process, more statistically robust. Gridded population sets such as LandScan and WorldPop can be loaded into GIS platforms, cropped to an administrative boundary of interest, and converted to a density grid. Spatial tools to ‘create spatially balanced points’ use a spatially interpolated algorithm to generate random points, naturally weighting the selection with more points selected in denser areas. The grid cells can then be ground-truthed for accuracy. A 2008 mortality study in Iraq used this technique and found it accurate, time-efficient and financially-feasible (Fig. 6 a and b) [29].

Humanitarian health surveys that require primary data collection are challenging. They are costly, time-consuming, take place in relatively dangerous and insecure (and thus inaccessible) environments, often failing to achieve the policy-changing impacts for which they were designed. In lieu of these, other geo-coded data sources exist in the humanitarian sphere (Table 1) that lend themselves to spatial analytics, reducing the time and cost to go from idea to evidence-based policy recommendation.

**Table 1:** Humanitarian geocoded data sources

Food and Agriculture Organization (FAO): geocoded data with global administrative layers. http://www.fao.org/geonetwork/srv/en/main.home	
UN Program on Global Geospatial Information Management, hosting agency for the UN Committee of Experts on Global Geographic Information which explicitly calls for a “stronger interoperability and integration between geospatial information and statistics” and for “combining statistics with spatial data” maintains a working group for such [10]. https://unstats.un.org/unsd/geoinfo/	
UN Spatial Data Infrastructure (UNSDI) maintains a portal for users from a wide range of disciplines, including humanitarian response http://geonetwork.nl	
Mapping units within other UN agencies: WHO, World Food Program, UNFPA	
UN High Commissioner for Refugees Data Portal: http://data.unhcr.org/imtoolkit/chapters/view/mapping/lang:eng	
Centre for Research and Epidemiology in Disasters (CRED) hosts the CEDAT database which includes geocoded nutrition and mortality data from humanitarian non-governmental organizations. http://www.cedat.be	
OpenStreetMap (OSM), the ‘wiki’ of geodata, open sourced. http://openstreetmapdata.com. ArcMap (ESRI) now has a tool to edit and publish within OSM	
Humanitarian Data Exchange: https://data.humdata.org	

The positivistic nature of empirical science emphasizes systematic methods of observation, measurement and evaluation. The adoption of geo-statistical science by humanitarian health researchers can only enhance empirical credibility. The opportunity to accurately measure, sample and apply inferential statistics and mathematically explain a level of significant geographic association or causality with a level of confidence and precision adds a new dimension to humanitarian health science, policy and programs.

Spatial epidemiology, a geographic variant on traditional epidemiology, is the logical framework for advancing geo-analytics in humanitarian settings [30]. By leveraging a validated field and adopting standardized semantics and methods, humanitarian health will enter a larger empirical conversation, improve its evidence base and evolve critical academic rigor. Despite the current paucity of experts that utilize spatial epidemiology for humanitarian health, many software programs and online communities exist to enable ‘non-experts’ to apply geospatial analysis for both research and operational pursuits (Table 2). The availability of user-friendly geospatial tools is key to addressing the current gap in expertise, but appropriate implementation of these tools requires an understanding of the fundamentals of spatial statistical theory.

**Table 2:** A sample of the geospatial analysis software programs available

ESRI Arc Software (https://www.esri.com/en-us/home): Includes ArcPro, ArcGIS, ArcGIS Online. A GIS for creating maps, compiling geographic data into geodatabases, geospatial analysis, visualization, and data sharing.	Subscription required
QGIS Software (https://qgis.org/en/site/): A GIS for creating maps, compiling geographic data into geodatabases, geospatial analysis and visualization.	Free and open source
SaTScan (https://qgis.org/en/site/): Software that analyzes spatial, temporal and space-time data using scan statistics.	Free and open source
R package SpatialEpiApp (https://cran.r-project.org/web/packages/SpatialEpiApp/index.html): A GIS for spatial epidemiological analysis that utilizes the R programming language and environment.	Free and open source
R package INLA (http://www.r-inla.org/): A software package within R that creates Bayesian models for spatial data.	Free and open source
STAN (https://mc-stan.org): Software for facilitating statistical inference that interfaces with many data analysis languages including R, Python, shell, MaTLAB, Julia, and Stata)	Free and open source

### Improving programmatic efficiency

Humanitarian crises and subsequent resource requirements are on the rise, yet funding continues to be insufficient -over ten billion U.S. dollars short as of UN OCHA’s 2018 projections [31]. It is thus imperative for humanitarians to determine the most cost-effective initiatives. We are beginning to see GIS tools and spatial analytics creep their way into other areas of public health and medical literature as a means to design, deploy, monitor and evaluate hyper-local, resource-efficient response. The growing evidence around geospatial analysis in health systems delivery, non-communicable diseases, hazard exposure, vector migration, communicable disease incidence, violent trauma, injury prevention, and econometric health indicators [32] should stand as examples for the humanitarian health community.

Health information and surveillance systems that prospectively track communicable and non-communicable diseases are now retrofitted with geocoded data to identify clusters and hot spots. Cluster analysis, a statistical method with which to identify hot spots, cold spots, spatial outliers, and similar features, is broadly applied in public health to target interventions ranging from Women, Infants, and Children (WIC) services [33] to naloxone distribution [34]. As social determinates of health become more acknowledged as lynch-pins for effective interventions, spatial dependence models placed within Bayesian hierarchical settings [35] provide flexible inferential frameworks to accommodate complex patterns of socioeconomic advantage and disadvantage in dynamic environments and create a proper assessment of uncertainty.

Geospatial analysis is evolving to describe, predict and model communicable, non-communicable and zoonotic diseases. Mechanistic spatial models are being developed and tested to predict the transmission of infectious diseases through individual-based simulation, meta-population models or network models [36].. The Global Epidemic and Mobility Model, validated by empirical surveillance during the 2009 H1N1 pandemic, uses a spatial layer of population distribution coupled with a transportation network layer and a flexible manipulated model of disease transmission to predict epidemic spread that is accurate against a gold standard [37]. Various geographically weighted regression models can now tell us where to deploy leptospirosis prevention programs [38], for example, with greater spatial resolution and resource efficiency. Scan statistics, hot spot analysis and Poisson kriging (forms of cluster analysis and interpolation, respectively) can highlight incidence clusters of cardiac arrest for intervention programs ranging from bystander CPR trainings to AED placement [39, 40].

**Fig. 7:**
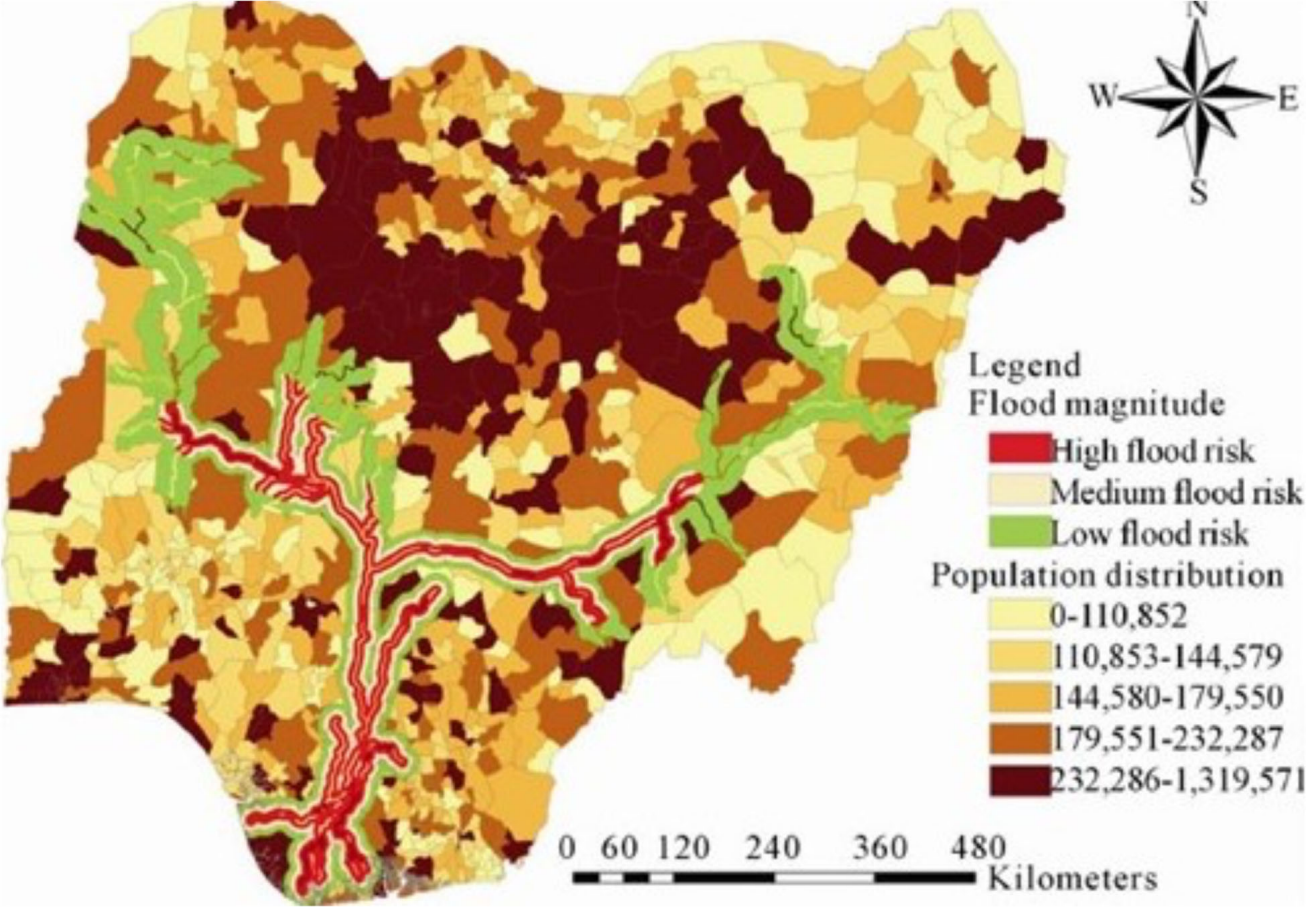
An example of hazard risk analysis: spatial distribution of population at risk to flood along the Niger-Benue river system, Nigeria. Multiple data layers of environmental features and historical flood zones are combined with population layers to synthesize flood risk for vulnerable populations. Terms of use: this work is licensed under Creative Commons Attribution License 4.0 and used unedited. Attributed to Nkeki, et al., JGIS, 2013; original version at: https://file.scirp.org/Html/3-8401214_29778.htm

‘Ecohealth’ frameworks that examine the link between humans, the environment and health, are identifying environmental, social and biological factors that impact both susceptibility and exposure to disease through the mathematic amalgamation of meteorology, demography, socioeconomics, and remotely sensed land use and topography data into a vulnerability index. These geospatial indices not only identify density of disease, such as dengue [41], but also elucidate the impact of each contributory variable, making programmatic intervention specifically targeted. Principal component analysis of environmental and sociodemographic variables is being linked to morbidity and mortality to create spatial vulnerability maps to address extreme heat events [42]. And in the glare of climate change, disaster managers are using models that characterize the increasing frequency of storms, temperature extremes, drought, sea level rise and storm surge for community vulnerability assessment and preparedness planning (Fig. 7) [43].

**Fig. 8:**
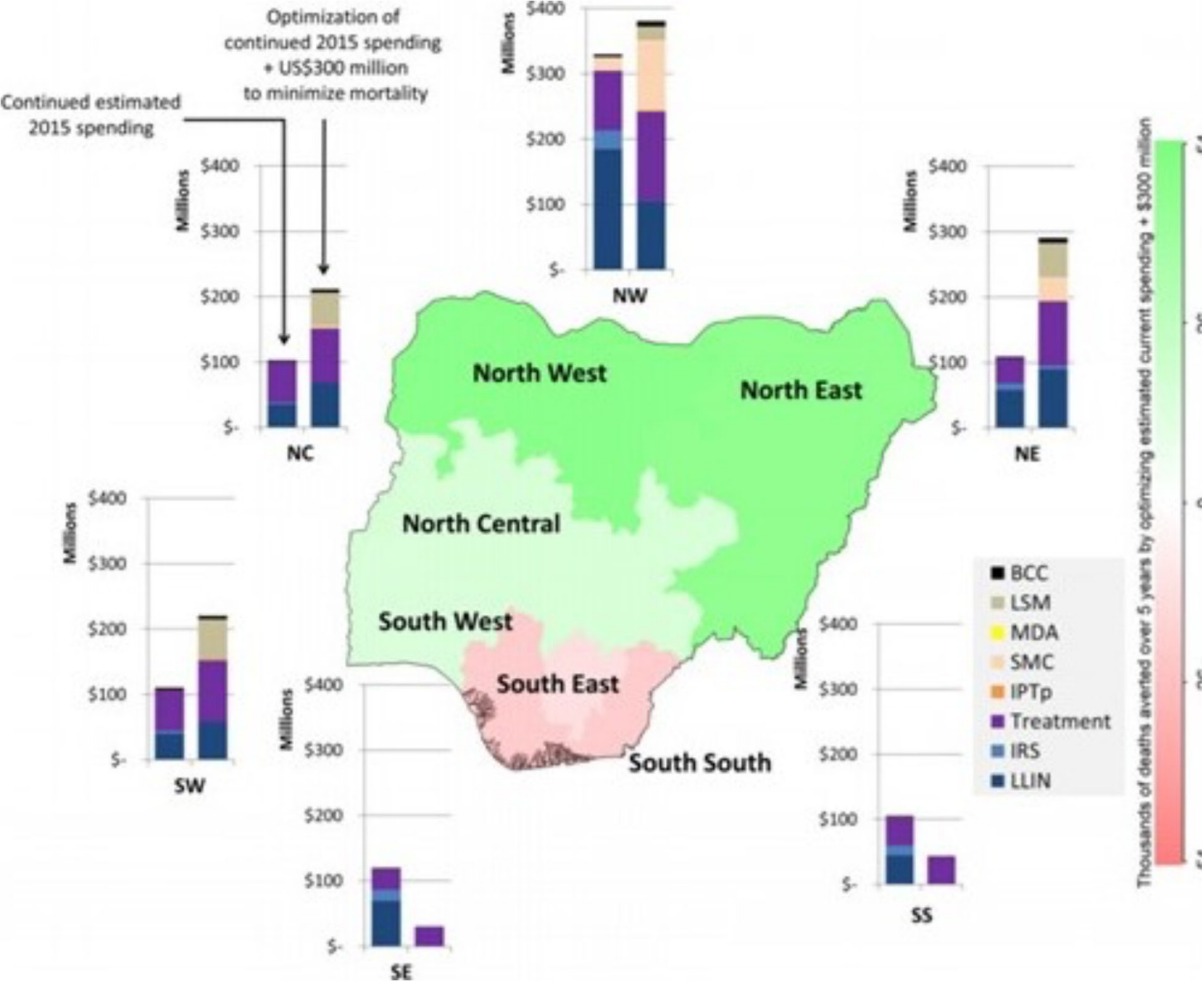
Geospatial optimization to minimize malaria mortality. Here a geospatial analysis of spending in health care for a range of malaria prevention and management in regions of Nigeria in 2015 can be modeled to estimate the number of deaths averted with additional funding. Terms of use: this work is licensed under Creative Commons Attribution License 4.0 and used unedited. Attributed to Scott, et al., Malar J, 2017; original version at: https://malariajournal.biomedcentral.com/articles/10.1186/s12936-017-2019-1

In a concerted effort to appreciate spatial heterogeneity and target quality improvement initiatives, health systems researchers, economists and administrators now embrace the spatial analysis of health care delivery, resource utilization and patient outcomes. Econometric models that utilize health care infrastructure variables as inputs and mortality as outputs are being used to understand the relationship between place, socio-economics and health system efficiency [44]. The use of a regression model that takes into account the spatial dependencies of error terms can highlight regional variations in types of ambulatory medical needs for system design purposes [45]. And most impressively, the incorporation of geographical optimization algorithms within disease distribution or epidemic models can estimate the optimal allocation of funding across population groups and programs. A seminal study utilized adaptive stochastic descent algorithms –a method that utilizes probabilistic assumptions to replicate aspects of the manual process of parameter fitting for optimization algorithms- to optimize malaria programs focused on reducing incidence and mortality in Nigeria (Fig. 8) [46]. Through allocative efficiency that prioritizes specific preventative and treatment activities in precise geographic locations, approximately 84,000 deaths or 15.7 million cases of malaria could be prevented over 5 years.

**Fig. 9:**
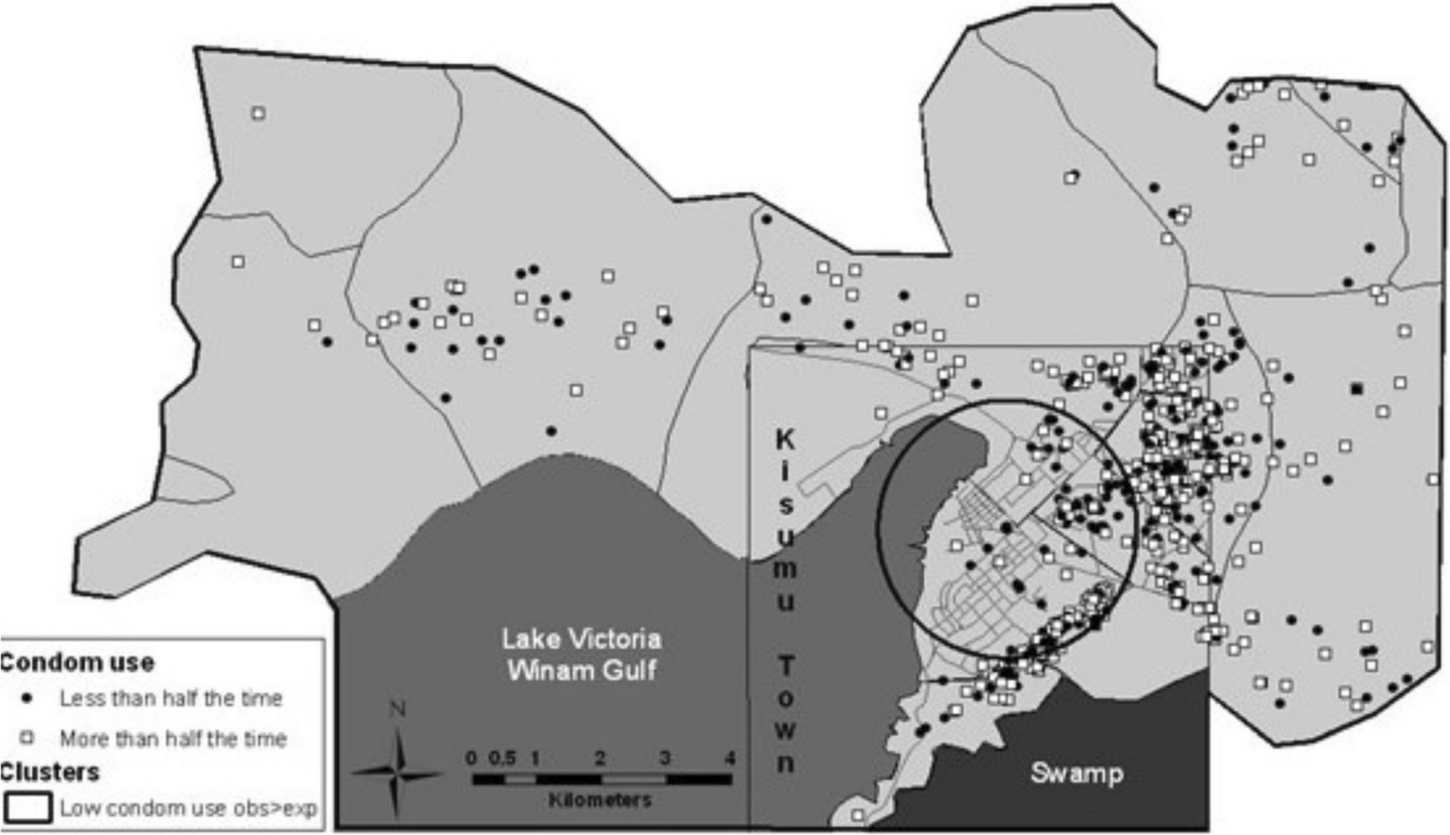
Cluster analysis of condom use in circumcised men at risk for HIV. Here, mapping program outcomes of an HIV prevention program (condom use) provides a basis for cluster analysis to better understand the reasons for success or failure of a health program. Used unedited by permission of the MEASURE Evaluation, University of North Carolina, Chapel Hill. Attributed to Moise, et al., 2015. Original version at: https://www.measureevaluation.org/resources/publications/ms-14-98

While there are a vast number of ways in which geospatial methods can inform programmatic design, spatial analytics can also improve monitoring and evaluation efforts. In secure environments, global health development projects have found geospatial analysis useful to identify geographic hot spots of programmatic success and to understand the spatially relevant variables that improve outcomes. Cluster analysis, using the Kuldorff scan statistic (Fig. 9), demonstrates how geo-tagged survey data can be used to monitor risk behaviors of recently circumcised men for HIV prevention efforts. Kernel density estimations, a non-parametric method for estimating the probability density function of a random variable, are effective for mapping catchment environments, given the spatial nature of care seeking behavior, and can show variability in access and medication supply [47]. Geospatial analysis can comment upon the success of vaccination centers during pandemics [48].

By sequentially running a time-series model of program coverage and a spatiotemporal Bayesian geostatistical model of infection, Bhatt et al. demonstrated the first formal quantification of *P. falciparum* infection prevalence, incidence and programmatic impact [49]. Data on insecticide-treated bed net (ITN) use, indoor residual spraying (IRS) and access to artemisinin-based combination therapy (ACT) from over one million households in sub-Saharan Africa were combined with *P. faciparum* parasite data from 27,573 georeferenced population clusters and environmental and sociodemographic covariates between 2000 and 2015. Not only were they able to attribute a prevention of 663 million clinical cases of malaria to interventions, but they were able to identify ITN coverage as the most important activity. Harnessing this potential of geospatial science for humanitarian health programs will revolutionize how interventions are conceived, designed and monitored, improving programmatic efficiency and impact.

## Conclusions

Geospatial data, the technology to capture and manage these data, and the science of geoanalytics are evolving at a spectacular pace in academia and industry. The geospatial lens has transformed ways of thinking about disease, the environment, socioeconomics and health. From environmental health research to disaster management, humanitarian coordination to logistics, geospatial analysis is being leveraged to ask novel questions, build academic rigor and improve programmatic efficiency. The humanitarian health community, with its empirical foundation and programmatic objectives, is primed for the adoption of geospatial analysis.

As evidenced, the plethora of diverse spatial frameworks and methods utilized in public health and humanitarian response, writ large, is nearly overwhelming. Thus, employing spatial analytics within the humanitarian health sphere will require cross-disciplinary collaborations and a strategic plan for the validation and standardization of specific and relevant geospatial constructs and methodologies. This argues for an increased role of academic centers with multiple but complementary disciplines that have advanced geoanalytic, mathematical, biostatistical and code-writing capabilities. These advanced skills and applications need to be couched within a humanitarian health framework to create workflows and outcomes that are translatable and actionable at the field level. While academic geospatial exploration in the name of humanitarian health is welcome, the goal for meaningful data mining and analysis will be to simultaneously optimize resources and save lives.

Geospatial analysis has exquisite power, but it too posits yet to be resolved ethical and data security questions. In this age of big, geo-tagged data, we often forget the power of that data, the biased infrastructures that obtain and utilize that data, and the very real danger of data insecurity. Conflict and disaster affected populations are fundamentally vulnerable. And humanitarian health data systems, although born from lofty intentions, have the potential to cause and magnify unique risks and harms that increase vulnerability and threaten dignity. A rights-based approach to data is critical, emphasizing the rights to data agency, protection, privacy and security [50]. Addressing these issues with intentionality and resources is crucial to the beneficent adoption of geospatial analysis.

The reality is that crisis epidemiology has refined the humanitarian health knowledge base and has built, over the years, through trial and error, the relevant domains and indicators to protect, address, and rebuild population health in crises. Nutrition, reproductive health, gender-based violence, mental health, communicable and non-communicable disease prevention and surveillance, to name a few, are all domains with spatial relevance. Enabling them with evidence-based, standardized, and ethical geostatistical methods will revolutionize humanitarian health, both academically and programmatically.

## Data Availability

Not applicable

## Abbreviations

API: Application programming interface
GIS: Geographic information systems
GPS: Global positioning systems
OCHA: UN Office for the Coordination of Humanitarian Affairs
UNITAR: UN Institute for Training and Research
UNOSAT: UNITAR Operational Satellite Applications Programme
VHR: Very high resolution
